# Differential Protein Expression in Exponential and Stationary Growth Phases of *Mycobacterium avium* subsp. *hominissuis* 104

**DOI:** 10.3390/molecules26020305

**Published:** 2021-01-08

**Authors:** Shymaa Enany, Manabu Ato, Sohkichi Matsumoto

**Affiliations:** 1 Department of Microbiology and Immunology, Faculty of Pharmacy, Suez Canal University, Ismailia 41522, Egypt; 2 Leprosy Research Center, Department of Mycobacteriology, National Institute of Infectious Diseases, Tokyo 189-0002, Japan; ato@niid.go.jp; 3 Department of Bacteriology, Niigata University Graduate School of Medicine, Niigata 951-8510, Japan

**Keywords:** *Mycobacterium avium* 104, proteins, proteomics, exponential, stationary, growth phases

## Abstract

*Mycobacterium avium* complex (MAC) is the most common non-tuberculous mycobacterium (NTM) and causes different types of pulmonary diseases. While genomic and transcriptomic analysis of *Mycobacterium avium* 104 (*M. avium* 104) has been extensive, little is known about the proteomics of *M. avium* 104. We utilized proteomics technology to analyze the changes in the whole proteome of *M. avium* 104 during exponential and stationary growth phases. We found 12 dys-regulated proteins; the up-regulated protein hits in the stationary phase were involved in aminopeptidase, choline dehydrogenase, oxidoreductase, and ATP binding, while the down-regulated proteins in the stationary phase were acetyl-CoA acetyltransferase, universal stress protein, catalase peroxidase, and elongation factor (Tu). The differently expressed proteins between exponential and stationary phases were implicated in metabolism and stress response, pointing to the functional adaptation of the cells to the environment. Proteomic analysis in different growth phases could participate in understanding the course of infection, the mechanisms of virulence, the means of survival, and the possible targets for treatment.

## 1. Introduction

Non-tuberculous mycobacteria (NTM) have less information compared to the classical mycobacterial pathogens such as *Mycobacterium tuberculosis* (*M. tuberculosis*) and *M. leprae*. The incidence of NTM diseases shows a dramatic rising over the past several years, especially in developed countries. In the United States, particularly among women and older age groups, the incidence has increased from 3.13 to 4.73 per 100,000 person-years from 2008 to 2015 [1]. NTM disease has reached 14.7 cases per 100,000 people based on national surveillance data in 2014 in Japan [2]. The prevalence of NTM lung disease appears to be increasing and is expected to overcome that of *M. tuberculosis* in the next few years [3].

The pulmonary *Mycobacterium avium* complex (MAC) disease is the most common in NTM diseases, responsible for over 80% of NTM diseases [4]. The pulmonary MAC disease is generated due to either *M. avium* subsp. *hominissuis* (MAH) or *M. intracellulare* and their prevalence differ from country to country. The prevalence of pulmonary MAC disease due to MAH is seven times more than that due to *M. intracellulare* [5]. The establishment of MAC diseases is associated with many factors; one of these factors is the bacterium themselves. To elucidate the mechanism of pathogenicity related to these bacterial factors that affect the establishment of MAC disease, many genomic and transcriptomic studies have been done. The complete genome of *M. avium* different subspecies and strains has been sequenced: MAH [6,7] and *M. avium* subsp. *paratuberculosis* [8]. However, the RNA sequencing for *M. avium* was performed for the transcriptome profiling [9,10]. Moreover, microarray was used in functional genomic research of *M. avium,* enabling a deeper analysis of their genomic diversity and an accurate measuring of most of their gene expressions [11].

While genomic and transcriptomic data have yielded a lot of information, the actual functional molecules have not been clearly elucidated. Proteomic study could determine functional protein networks that exist in the cell. Most previous studies of *M. avium* proteome was used to detect protein expression patterns in response to a specific stimulus like antibiotic usage [12], different environmental conditions [12], or under conditions mimic the infectious stages [13]. Comparative gene expression in-between strains or even in a single strain grown under different circumstances has confirmed its usefulness in recognizing mycobacterial growth and its pathogenicity determinants [14]. These studies were mainly focused on the identification and characterization of the cell wall associated proteins of *M. avium* [15] or their secreted proteome [13]. To our knowledge, investigation of the whole proteome of *M. avium* under different growth phases has not been elucidated yet. It is almost established from former studies on model organisms such as *Escherichia coli* and *Bacillus subtilis* that the transition from the exponential phase to the stationary phase is associated with changes in the expression patterns of proteins [16,17,18].

Little is known about the protein expression in MAH 104 during exponential and stationary growth phases and how this change contributes to pathogenesis. Here, we harness the proteomics technology to analyze the whole proteome of MAH 104 in these two growth phases. This will aid in better understanding the MAH 104 infection course and will help in its treatment. Particularly when the MAH 104 infection treatment period is long to very long, it is associated with mediocre to good outcomes. Therefore, basic knowledge of the cell biology is needed to develop new treatment strategies. Knowing the exact protein expression pattern might help to identify potential drugs targets.

## 2. Results

**Figure 1:**
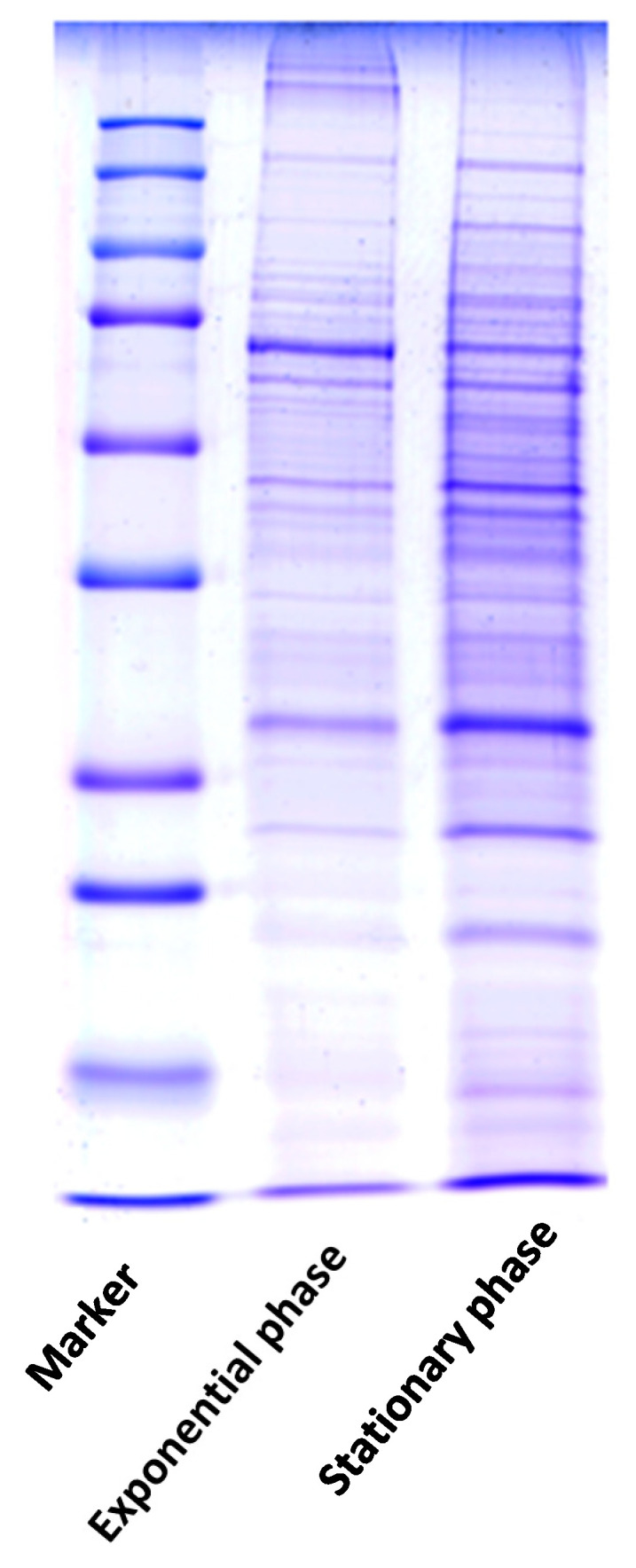
Coomassie Brilliant Blue R-250 stained gel image of protein extracts of exponential growth phase (lane 2) and stationary growth phase (lane 3) of MAH 104 from cells grown to OD600 = 0.5 and 2.8, respectively. Samples were run on 12.5% SDS-PAGE.

**Figure 2:**
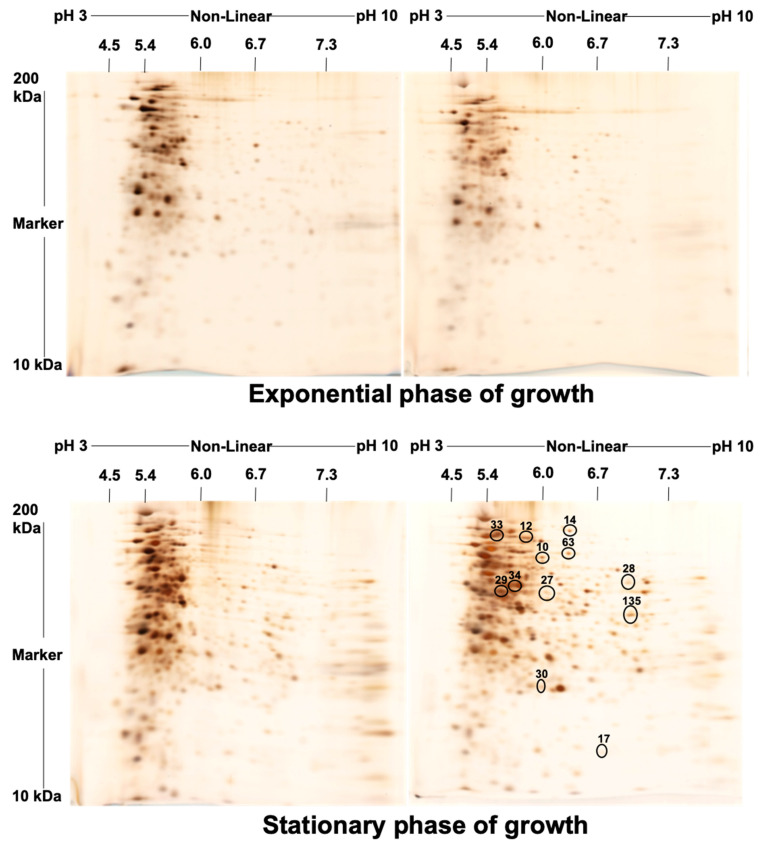
Duplicate sets of two-dimensional gel electrophoresis using immobilized pH gradient strips. pH 3–10 NL for proteins extracted from the exponential and the stationary growth phases of MAH 104. The horizontal axis represents the isoelectric point (pI) ranging between 3–10 and the vertical axis represents the second-dimension electrophoresis by molecular weight. Gels were silver-stained. The 12 significantly differentially expressed spots (≥2-fold change) between the exponential and the stationary growth phases that applied to nLC-MS/MS analysis were marked by circles.

The two samples of MAH 104 in exponential and stationary growth phases showed a relatively similar fractionation pattern on Coomassie Brilliant Blue stained SDS-PAGE gels with a characteristic variation in the intensity of some bands (Figure 1). For better resolution, we utilized two-dimensional electrophoresis (2-DE) with isoelectric focusing of isoelectric non-linear (pI 3–10) as the first dimension. Duplicate 2-DE images of silver-stained gels for proteins extracted from exponential and stationary growth phases are shown in Figure 2. The proteome patterns generated from each phase represent well-resolved protein spots.

Progenesis SameSpot software (Totallab) initially identified 518 dys-regulated spots in the gel images of the extracted protein from the stationary phase compared to that of the exponential growth phase. Initial analysis of these spots was carried out, as appeared in the chart in Supplementary Figure S1. Filtering was done to eliminate biased false positives spots produced from staining artifacts. Filter 1 (F1) was based on the spot volume intensity to keep spots significantly different by ≥2 fold. Filter 2 (F2) was according to the ANOVA to select the spots significantly different between the two-phase gels (*p* ≤ 0.05). Filter 3 (F3) was a manual one to remove all the din spots. The overlapping differentiation using the former filtering criteria among the gels of the two phases resulted in the final identification of 12 differentially significant spots with over two-fold difference between the two phases of growth (Supplementary Figure S1). As the results, the 12 selected spots are shown and marked by circles in a representative gel image for the proteins of the stationary growth phase (Figure 2). These significantly different spots were then selected and excised from the gel for mass spectrometric identification.

**Figure 3:**
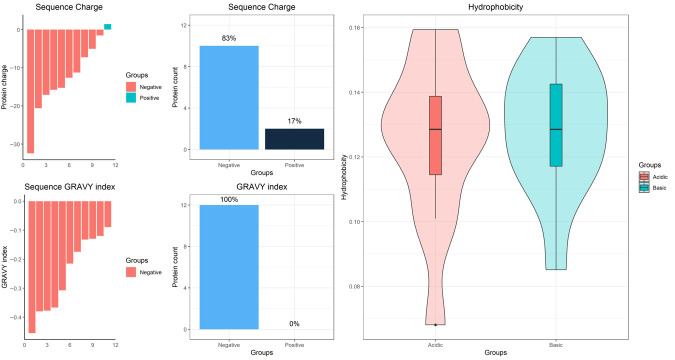
Physicochemical properties of the differentially expressed proteins between the exponential and the stationary growth phases of MAH 104. The panel is generated by the function PlotPhysicochemical () showing the distribution and ratios of sequence charge, GRAVY index, and hydrophobicity.

**Figure 4:**
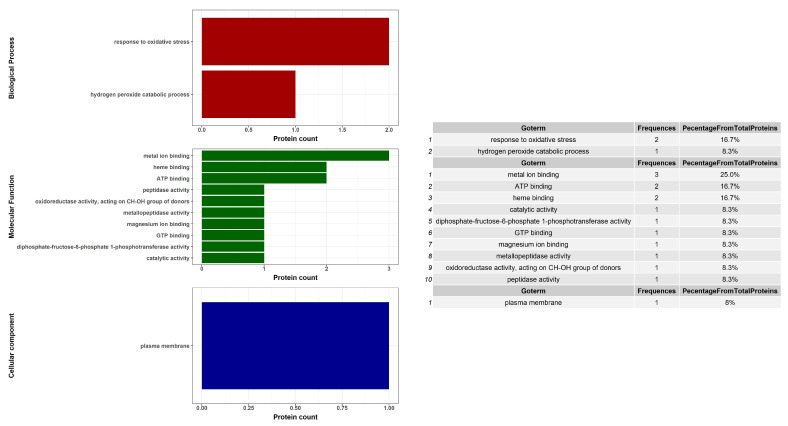
Gene ontology (GO) analysis and biological pathways of the differentially expressed proteins between the exponential and the stationary growth phases of MAH 104. Bar plots shows 12 enriched GO terms of molecular function, biological processes, and subcellular localization generated by the function PlotGoInfo.

Progenesis SameSpot software revealed eight proteins were up-regulated in the stationary phase, while the other four proteins were down-regulated. The software analysis revealed that the intensities of the targeted spots were differentially expressed between the two growth phases, as appeared in the gel image (Supplementary Figure S2A) and in the 3D views (Supplementary Figure S2B). This was confirmed by the logarithmic normalized volume between the two phases that blotted for each spot by SameSpots (Supplementary Figure S2C). For instance, the upper line is for spot number 63 (Glucose methanol choline) that is up regulated in the stationary phase and the lower one is for spot number 17 (Universal stress protein), which is down regulated in the stationary phase (Supplementary Figure S2 and Table 1). Average normalized volumes of the twelve differentially expressed proteins and the fold change values between each spot in the two phases were summarized in Table 1. The standard expression profiles of all the 12 dys-regulated spots differentially expressed between the two phases of growth of MAH were shown in Supplementary Figure S2D. The 12 spots were then identified by LC-MS/MS and 12 hits were detected with high confidence (Mascot score ≥ 30; FDR < 1%) and with protein scores ranging from 323 to 2605. Physiochemical properties of the significantly different proteins like the distribution and ratios of sequence charge, GRAVY index, and hydrophobicity are shown in Figure 3. Further annotation of these proteins with regard to their molecular function and biological process were searched. Gene Ontology (GO) analysis and biological pathways identification are done to reveal the biological process and pathways for the differentially expressed proteins (Figure 4). The identified up-regulated protein hits were involved in different processes like the aminopeptidase, choline dehydrogenase, oxidoreductase, and ATP binding. Mass spectrometric identification of the down-regulated proteins revealed acetyl-CoA acetyltransferase, universal stress protein, catalase peroxidase, and elongation factor (Tu). Their functions were involved in transferase activity, responses to the stress, the process of hydrogen peroxide catabolic with responses to the oxidative stress, and the activity of GTPase with the translation elongation factor activity, respectively (Table 1).

**Figure 5:**
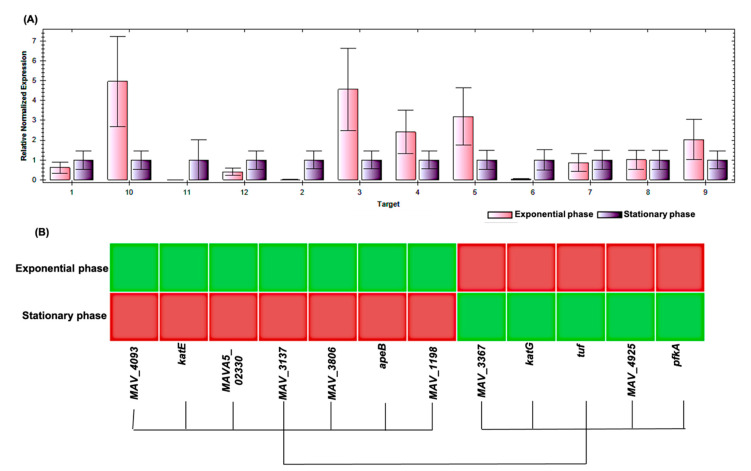
Analysis of expression of the twelve genes in the exponential and in the stationary growth phases by qRT-PCR. Cultures were grown to an OD600 of 0.5 and 2.8, respectively, and the total RNA was extracted from each phase. (**A**) mRNA levels were normalized using the geometric mean of the housekeeping genes, *sigA* and *groL*. Data are shown as the means of triplicate experiments with three biological replicates. (**B**) Heatmap for the differential expression of the 12 genes between the exponential and the stationary phase of the growth of the MAH 104. Gene numbers correspond to those listed in Table 1.

The genes responsible for the production of the differential proteins are shown in Table 1. They were detected by traditional PCR. Amplicon sizes for each gene products are summarized in Supplementary Table S1. The intensities of the band on the agarose gel were relatively different between the exponential and the stationary phases of growth (Supplementary Figure S3). For further confirmation and to investigate the dys-regulation at the transcription level, we performed quantitative reverse transcription polymerase chain reaction (qRT-PCR). RNA quality was assured, as appeared on the gel (Supplementary Figure S4A), and as shown in the spectrometer results that gave high purity with reasonable concentrations (Supplementary Figure S4B). RNA integrity was promoted using Bioanalyzer; RNA Integrity Number (RIN), the quality measurement from Agilent Technologies was 9.4 and 9.1 for RNA from MAH 104 in exponential and in stationary growth phases, respectively. Electropherogram profiles for RNA resolved on a Pico Chip of Agilent Bioanalyzer were accurate (Supplementary Figure S4C,D). Expression degrees were detected by the comparative threshold cycle through normalization to *sigA* and *groL*. Transcription analysis revealed a down-regulation in the mRNA of elongation factor (*tuf*) (5 fold), ATP binding protein (*MAVA5_02330*) (4.6 fold), universal stress protein (*MAV_3137*) (2.6 fold), dibenzothiophene desulfurization enzyme C (*MAV_3806*) (3 fold), and catalase (*katE*) (2 fold) (Figure 5A). The expression of other genes was up-regulated at the transcriptional level, as shown in Figure 5A, and in the heatmap (Figure 5B). Changes in RNA levels were rather different to the changes in protein levels.

The consistency between the transcriptomic and proteomic results for each differentially expressed hit were illustrated in Supplementary Figure S5 as a correlation between the average normalized volume of each protein and the quantitation cycle (Cq) mean for each gene. Red circles showing the hits that were up-regulated at both transcriptomic and proteomic levels, green circles showing the hits that were down-regulated at both transcriptomic and proteomic levels, and black circles showing the differently expressed hits between the transcriptomic and proteomic levels. The number of hits in Supplementary Figure S5 is the same number of genes and proteins hits in Table 1.

## 3. Discussion

As there are well-known disparities between cells in exponential and in stationary growth phases at the transcriptomic level, it is crucial to monitor and compare the changes in the proteome profile of *M. avium* through these two different growth phases. Former studies revealed remarkable growth phase associated alterations in protein expression within *M. smegmatis* [19] and *M. tuberculosis* [20]. In our study, mono-dimensional electrophoresis for the proteins in two phases, not unexpectedly, showed variations in some band intensities between samples as same protein could be identified in two different growth phases and up/or down regulated according to the phase. Simultaneously, two-dimensional gel electrophoresis, coupled with mass spectrometric and bioinformatic analysis, as a gel-based proteomics approach, showed that the expression of 12 proteins altered in the stationary phase. 

As expected, we found that elongation factor Tu was down regulated in the stationary growth phase. Notoriously, this protein has translation elongation activity and plays a significant role in cell energy metabolism, which is not a critical point in the stationary phase [21]. Elongation factor Tu has been known for its roles in protein synthesis, in adherence, and in immune regulation [22]. In a similar fashion, a protein, which is related to the transferase activity, has been declined in the stationary phase, since it is known that the transferase activity is needed for maintaining energy inside the bacterial cell, which is again less necessary during this growth phase [21,23]. Our results were consistent with what was previously established for *E. coli*, which are proteins associated with energy metabolism and phosphotransferase proteins are down-regulated through the stationary growth phase relative to the exponential growth phase [24].

On the other side, eight proteins were up-regulated in the stationary growth phase; some of these proteins were involved in maintaining osmotic balance of the living cells under stress such as glucose-methanol-choline and dibenzothiophene desulfurization enzyme C, which have oxidoreductase activity. This result was previously confirmed at the transcriptomic level of *E. coli,* which revealed up-regulation of genes that are involved in survival during osmotic stress [25].

Another up-regulated protein that was identified in our proteomic analysis was ATP-dependent Clp protease, suggesting its contribution to the stationary phase survival by controlling protein quality. ATP-binding protein, which is pertinent to the anaerobic respiration process, is required after entering the stationary growth phase. This is concordant with a previous study performed on *M. smegmatis,* showing that genes involved in anaerobic respiration have been up-regulated in the stationary phase [21]. Another protein obliquely involved in the anaerobic respiration and noted to be up regulated in our study is 6-phosphofructokinase 1, which aids in the conversion of fructose 6-phosphate and ATP to fructose 1,6-bisphosphate and ADP as a critical step of glycolysis that is deemed to be the base for both anaerobic and aerobic respiration. Interestingly, we found that one of the up-regulated proteins in the stationary phase, LprG protein, was associated with bacterial virulence and antibiotic resistance [26] and this is unsurprising since the secreted virulence factors of most human pathogens are often increased in the stationary phase of growth [27]. It has been proved that LprG involved in triacylglyceride levels, growth rate, and virulence regulation [28]. Its implication in mycobacterium virulence has appeared through the induction of mitochondrial fission, interference with complex I and complex II respiration, and modification of mitochondrial calcium uptake, which in turn proposes that LprG-stimulated cells are in a lower bioenergetics state, which could support its immunosuppressive capacity in infection [29]. The identification of LprG protein in *M. tuberculosis* was previously confirmed as P27 in the Mycobacterium tuberculosis complex and its gene is conserved across several pathogenic and nonpathogenic Mycobacterium species [30].

Aspartyl aminopeptidase, a protein that has a role in the protein turnover, was up regulated in the stationary phase in our study. It was established that during the exponential phase, bacteria degrade l–2% intracellular protein, while during stationary phase, it degrades 5–12% [31]. These alterations could be attributed to the nutrient limitation during the stationary phase; thus, the degradation of certain proteins becomes a demand in order to confer the amino acids required for the syntheses of a new protein [31].

A recent study has sequenced highly transformable virulent MAH approving novel genes that are essential for infection establishment. From these genes, some were involved in the growth and others in the virulence of the bacteria [32]. *katG* and *pfkA* were of the genes that encodes for catalase peroxidase and phosphofructokinase, respectively, which were down and up-regulated in the stationary phase in our proteomic analysis, respectively. Catalase peroxidase has a critical role in mycobacterial pathogenesis, it is countering the phagocyte oxidative burst and it has been shown to protect *M. tuberculosis* against the reactive oxygen intermediates in macrophage since catalase and peroxidase activities are associated with survival inside macrophages [33]. However, phosphofructokinase is implicated in the first committing step of glycolysis, which irreversibly catalyzes the phosphorylation of fructose-6-phosphate to fructose-1,6-bisphosphate. Phong et al. demonstrated that phosphofructokinase is essential for mycobacterial growth on glucose as a sole carbon source, and is responsible for the total phosphofructokinase activity in *M. tuberculosis* [34].

These changed protein expression levels that have been proved in our study between the two growth phases might, for most of our identified proteins, underlie functional adaptations of the cells, such as raising in cell synthesis and metabolism during the exponential phase, while elevating in the virulence and increasing adaptation, as well as survival in the stationary phase. However, there were some controversial results. Thus, we performed transcriptomic analysis to unravel the discrepancy.

Previous transcriptomic studies of *M. avium* have been focused on transcriptome during infection [10] or during the mid-exponential growth phase [9]. We explored the expression of the dys-regulated proteins that were detected by proteomic analysis between the two phases at the transcriptional level. Our results showed a down-regulation of elongation factor and universal stress protein coherently with the proteomic results. Strikingly, universal stress protein was down- regulated at both levels, although previous study proved the up-regulation of genes that are implicated in responses to stress in *E. coli* in stationary growth phase [25]. This could be attributed to the notion that responses to alterations in resource availability could vary between species [35]. Alternatively, the transcription of acetyl-CoA acetyltransferase in the stationary phase was down-regulated even when it had a 2.2 fold increase at the proteomic level. Additionally, ATP-dependent Clp protease ATP-binding protein and dibenzothiophene desulfurization enzyme C were down-regulated at the transcriptomic level during the stationary growth phase, although they were up-regulated at the proteomic level. These differences between transcriptome and proteome over the two growth phases could be attributed to several factors like the extremely low abundance of transcripts and the poor recovery of proteins due to its low solubility or the membrane attachment. Additional factor is the fact that a single transcript can be translated several times to protein, and protein is more stable than transcript, thus accumulating more than transcripts [36]. Another likely explanation for the transcriptome and proteome differences is post-transcriptional regulation. This slight difference between the transcriptomic and the proteomic results were also confirmed in our correlation graph (Supplementary Figure S5). The weak correlation between the transcriptome and the proteome implies a major role for regulation at the post-transcription level in growth phase adaptation [36].

## 4. Methods

### 4.1. Bacterial Strains, Culture Media, and Growth Conditions

The *M. avium* (MAH 104) strain was grown in Middlebrook 7H9 broth (Difco), supplemented with 0.2% glycerol, ADC, and 0.05% Tween 80 (MP Biomedical, LLC (Solon, OH, USA). When *M. avium* grows to OD_600_ = 0.5, this is considered as the exponential phase and when it grows to OD_600_ = 2.8, this is considered to be the stationary phase. Each measurement was detected from three biological and three technical replicates.

### 4.2. Bacterial Protein Preparation

Cells were harvested by centrifugation at 4000× *g* at 4 °C for 10 min. Cellular proteins were obtained using bead beating in a lysis solution (500 mM Tris pH 7.0, 130 mM DTT, protease inhibitor cocktail and 4% *w*/*v* SDS) and acetone [37]. Cellular debris was eliminated by centrifugation and protein lysate was collected. Proteins used for 2-DE analysis were re-suspended in 2-DE rehydration lysis buffer, while others were re-suspended in water. Bicinchoninic acid (BCA) assay kit (Pierce, Thermo Scientific, Rockford, IL, USA) was utilized to detect protein concentration using bovine serum albumin as a standard [38]. Each measurement was detected from three biological and three technical replicates.

### 4.3. SDS-PAGE

Six micrograms of protein extract for each sample were run on 12.5% SDS-polyacrylamide gels [39]. The gel was stained with Coomassie Brilliant Blue stain (CBB R-250, Wako, Saitama, Japan).

### 4.4. Isoelectric Focusing (IEF) and Second Dimension Gel Electrophoresis (2-DE)

First dimensional IEF was performed using Ettan IPGphor 3TM (Amersham Biosciences, Buckinghamshire, UK) with 7 cm ImmobilineTM DryStrip Non-Linear both pH 3–10 and pH 4–7 (GE Healthcare, Amersham, UK). Rehydration of the gel strips was done according to the manufacturer’s instructions. Protein extracts were further diluted in Destreak rehydration buffer and were focused up to 9400 V·h at a maximum voltage of 5000 V (step and hold). Following IEF, proteins were reduced and alkylated by soaking the Immobiline Drystrips in 5 mL equilibration solution (6 M urea, 50 mM Tris HCl; pH 8.8, 30% glycerol, 2% SDS, 0.004% bromophenol blue) containing 50 mg dithiothreitol for 15 min at room temperature and then in 5 mL equilibration solution containing 125 mg iodoacetamide with the same conditions [40]. Equilibrated strips were directly used for the second dimensional SDS-PAGE, as described by O’Farrell [41]. Strips were sealed on top of the SDS-PAGE gel (12.5% polyacrylamide) with 0.5% agarose. Tris glycine SDS (25 mM Tris, 198 mM glycine, and 0.1% SDS) was used as electrode buffer. Gels were run at 20 mA/gel until the dye front reached the bottom edge of each gel. The 2-DE was run in duplicate for each sample.

### 4.5. Staining of the Gel and Visualization of the Spots

Silver stain was used to stain the gels (2D-Silver Stain Reagent II, Cosmobio Co., Ltd., Tokyo, Japan) according to the manufacturer’s instruction. For gel imaging, the 2-DE protein patterns were recorded as digitalized images using a high-resolution scanner. Images were captured at 800 dpi as TIEF file for comparison. Scanned TIEF images were compared using Progenesis SameSpots software (Totallab, Newcastle, UK) to detect the significantly different spots between gel images. Briefly, SameSpot overlaid images and grouped them according to the predefined settings. Next, the landmark spots were automatically defined and confirmed manually. We selected a reference image where other images were normalized on it (usually the one contains the maximum number of spots). In a final step, the software calculated the intensities of all sots of the reference image and normalized other images based on that one. Significant spots were selected with ANOVA with *p* ≤ 0.05 and cutoff spots ≥2-fold different. These spots were then cut out for mass spectrometric exploration.

### 4.6. Processing of the Spot and Mass Identification (TripleTOF MS/MS)

The selected spots were cut out and subjected to de-staining using Silver Stain Kit for Mass Spectrometry^®^ (Pierce, Thermo Scientific, Waltham, MA, USA) to facilitate the complete removal of silver from stained protein spots and maximum protein recovery for subsequent mass spectrometry analysis. Each spot was reduced with 10 mM dithiothreitol, carbamide methylated with 55 mM iodoacetamide, and treated with trypsin for digestion. The peptides were dissolved in 0.3% formic acid and injected to the nano-flow-LC (Eksgent nanoLC 415 with ekspert cHiPLC, AB Sciex, Framingham, MA, USA) with the tandem MS (TripleTOF5600, AB Sciex). Duplicate analysis was performed in trap and elute mode of the ChromeXP C18 Chip column (200 μm × 0.5 mm) and the ChromeXP C18 Chip column (75 μm × 150 mm). Formic acid (0.1%) was used as a mobile phase A and formic acid (0.1%) in acetonitrile as a mobile phase B. Acetonitrile was used as an organic solution to extract hydrophobic peptides to optimize the peptide extraction. Elution of 20-min gradients from 2% B to 32% B were done. Mass spectrum in the data dependent mode was performed. 

### 4.7. Bioinformatic Analysis

Our outputs were examined against the *Mycobacterium avium* 104 UniProt KB database (Proteome ID UP000001574) with concatenated decoy database using Mascot search engine (version 2.4, Matrix Science, London, UK). If the peptides swept the identity and homology thresholds of the Mascot algorithm, it takes up with FDR <1%. Gene Ontology annotations were explored from the UniProt database (http://www.uniprot.org/).

### 4.8. Traditional PCR Reaction

The 12 genes responsible for the production of the 12 differential proteins identified between the two phases were detected by PCR. The amplified products were analyzed in a 2.5% agarose gel stained with ethidium bromide and visualized in UV light. PCR amplicon of each band was determined by comparison to the corresponding band in the low molecular weight ladder. Primers for 12 genes were outlined using the publicly available Primer3 software, as shown in Supplementary Table S1.

### 4.9. RNA Extraction

RNA was extracted from the two samples using TRIzol (Invitrogen, Carlsbad, CA, USA) according to the manufacturer’s instructions. Cells were precipitated by centrifugation at 4000× *g* and were re-suspended in 1 mL TRIzol, followed by disruption. The samples were incubated with chloroform and the pellets were dried and then re-dissolved in ultrapure RNase free water (Invitrogen). The quantity and the quality of RNA were checked by gel, spectrometer, and bioanalyzer.

### 4.10. Quantitative Reverse Transcription Real-Time PCR (qRT-PCR)

ReverTra Ace qPCR RT Master Mix with gDNA Remover (Toyobo, Osaka, Japan) were used, following the manufacturer’s recommendations for the synthesis of the cDNA. The reaction was carried out using THUNDERBIRD SYBR qPCR Mix (Toyobo) following the manufacturer’s instructions. The same primers used in the traditional PCR were used together with these primers as internal standards; 5′-CGTTCCTCGACCTCATCCA-3′ and 5′-GCCCTTGGTGTAGTCGAACTTC-3′ (for *sigA*); 5′-ATTGCGTATGACGAAGAGGCCCG-3′ and 5′-TTCTTCTCCAGGACGACGTTGCG-3′ (for *groL*) [42,43]. The reactions were done with the CFX Connect Real-Time System (Bio-Rad). Relative gene expression was detected from a calculated threshold cycle (CT) and then normalized versus *sigA* and *groL* as internal standards. Our reaction was performed for three biological and three technical replicates.

## 5. Conclusions

Ultimately, our proteomic analysis revealed that the differences between exponential and stationary phases are accompanied by variations in the gene expression. The different proteins were implicated in metabolism, virulence, adaptation, and surviving of bacteria pointing to the functional adaptation of the cells to the environment. For most of our identified proteins, differences in protein expression levels between the two growth phases underlie functional adaptations of the cells such as raising in cell synthesis and metabolism during the exponential phase, while elevating in the virulence and increasing adaptation as well as survival in the stationary phase. Studying *M. avium* pathogen at the proteomic level in different growth phases could participate in understanding the course of infection, the mechanisms of virulence, the means of survival, and the possible targets for treatment. 

## Figures and Tables

**Table 1:** Average normalized volumes and the functional identification of the significantly differentially expressed proteins identified by nano-LC-MS/MS between the exponential and the stationary growth phases of *Mycobacterium avium* 104.

Spot No.	Fold Change (+/−) *	ANOVA (*p*)	Average Normalized Volumes		Accession No.	Protein Name	Protein Score	No. Identified Peptides	Gene Name	No. (qRT-PCR)	Molecular Function	Regulation at Transcriptomic Level *
			Exponential Growth Phase	Stationary Growth Phase								
10	+4.4	0.018	11,896.756	52,584.334	A0A0H2ZX69	Pyruvate decarboxylase	655	25	*MAV_4093*	1	Catalytic activity	+
12	+4.3	0.038	4334.274	18,578.945	A0A0H2ZZB0	Catalase	2211	21	*katE*	2	Catalase activity	+
14	+4	0.048	8140.139	32,853.588	A0A0E2WBQ0	ATP-dependent Clp protease ATP-binding protein	1176	24	*MAVA5_02330*	3	ATP binding and Chaperone	−
17	−3.2	0.035	33,720.648	10,671.303	A0A0H3A465	Universal stress protein family protein	348	24	*MAV_3137*	4	Response to stress	−
27	+2.2	0.003	7519.716	16,866.019	A0A0H2ZYK5	Dibenzothiophene desulfurization enzyme C	353	21	* MAV_3806 *	5	Oxidoreductase activity	−
28	+2.2	0.045	9994.532	21,965.532	A0A0H2ZU78	aspartyl aminopeptidase	419	26	* apeB *	6	Aminopeptidase, Hydrolase, Metalloprotease, and Aminopeptidase activity	+
29	−2.2	0.046	530,650.636	244,551.945	A0A0H2ZT78	Acetyl-CoA acetyltransferase	1532	26	*MAV_1198*	7	Transferase activity	+
30	+2.1	0.029	19,961.352	42,368.898	A0A0H3A4W6	LprG protein	323	26	* MAV_3367 *	8	Phosphatidylinositol binding	+
33	−2	0.035	449,155.112	221,267.396	A0QGA4	Catalase-peroxidase	2066	25	* katG *	9	Catalase activity	−
34	−2	0.015	150,556.978	75,806.224	A0QL35	Elongation factor Tu	2605	20	*tuf*	10	GTPase activity + translation elongation factor activity	−
63	+5.1	0.050	6531.054	33,156.744	A0A0H2ZRA7	Glucose-methanol-choline	1014	26	* MAV_4925 *	11	Choline dehydrogenase activity	+
135	+2.2	0.050	35,082.949	77,486.389	A0A0H3A360	6-phosphofructokinase 1	800	23	* pfkA *	12	6-phosphofructokinase activity	+

* Regulation in stationary phase; + is up regulated and − is down regulated.

## Data Availability

No new data were created or analyzed in this study. Data sharing is not applicable to this article.

## Supplementary Materials

The following are available online, Figure S1: The workflow of our gel spot refining strategy. Three filters (two automated and one manual) were used for refining Progenesis SameSpot selected spots. F1 is a spot volume intensity filter to identify spots different by ≥2-fold. F2 is statistical ANOVA filter to identify spots significantly different at *p* ≤ 0.05. F3 is manual filtering to eliminate suspected background and noise spots. Visualization was achieved by silver staining before spot picking and was followed by LC-MS/MS analysis of spots, Figure S2: Gel spot comparison for two representatives differentially expressed proteins between the exponential and the stationary growth phases of MAH 104 using Progenesis SameSpots software; Upper line for spot number 63 (Glucose methanol choline) that is up regulated in the stationary phase and lower line for spot number 17 (Universal stress protein) that is down regulated in the stationary phase. Intensities of the two spots differentially expressed in the exponential and the stationary growth phases were shown in the representative gel image (A). 3D views of the intensities of the targeted spots blotted by SameSpots in both phases are shown (B). The logarithmic normalized volume between the 2 phases was blotted for each spot by SameSpots (C). Spot numbers are corresponding to those listed in Table 1. (D) The standard expression profiles of the twelve differentially expressed proteins between the exponential and the stationary growth phases of MAH 104, Figure S3: Agarose gel electrophoresis of 12 PCR products of genes responsible for the expression of the 12 differentially expressed proteins between the exponential and the stationary growth phases of *Mycobacteriun avium* 104. Low molecular weight markers are used. Band sizes for each gene product of the 12 genes are summarized in Table 1, Figure S4: *M. avium* RNA in exponential and in stationary growth phases quality on the gel (A), purity and concentrations using eppendorf Bio-spectrometer (B) and integrity; electropherogram profiles for *M. avium* RNA resolved on a Pico Chip of Agilent 2100 Bioanalyzer, Agilent Technologies (C). (D) Gel image formed for *M. avium* RNA using Agilent 2100 Bioanalyzer, Agilent Technologies, Figure S5: Correlation between the gene expression of each gene and the average normalized volume for each protein of the differentially expressed hits between the two growth phases. Numbers shown here indicating the number of genes and proteins as displayed in Table 1. Red circles; up-regulated, Green circles; down-regulated, Black circles; differ in/between the transcriptomic and the proteomic analysis, Table S1: List of primer sequences used in this study for 12 genes that were designed with the publicly available Primer3 software.

## Sample Availability

Samples of the compounds are not available from the authors.
