# Supplementary material for: Differential Protein Expression in Exponential and Stationary Growth Phases of Mycobacterium avium subsp. hominissuis 104
Source: Molecules. 2021 Jan 8;26(2):305. doi: 10.3390/molecules26020305 (PMC7827551; doi:10.3390/molecules26020305)
Supplement: Supplementary file 1 [file molecules-26-00305-s001.pdf]

Supporting Information

Title:

Differential Protein Expression in Exponential and Stationary growth phases of *Mycobacterium avium* subsp. *hominissuis* 104

Authors:

Shymaa Enany[1]*, Manabu Ato[2], and Sohkichi Matsumoto[3]*

Affiliations:

[1]Department of Microbiology and Immunology, Faculty of Pharmacy, Suez Canal University, Egypt.

[2]National Institute of Infectious Diseases, Department of Mycobacteriology, Leprosy Research Center, Tokyo, Japan.

[3]Department of Bacteriology, Niigata University Graduate School of Medicine, Niigata, Japan.

*Corresponding authors e-mails: shymaa21@yahoo.com & sohkichi@med.niigata-u.ac.jp

ORCID; Enany: 0000-0002-7827-6504 & ORCID; Matsumoto: 0000-0002-6106-7538

Table S1. List of primer sequences used in this study for 12 genes that were designed with the publicly available Primer3 software.

Gene name	Primer name	Primer sequences (5′-3′)	Product size (bp)
MAV_4093	1_F<br>1_R	CCATCAAGGATCCCGACGAG<br>ACTTGAACTGCTCCCAGGTG	135
katE	2_F<br>2_R	TCAAGTTCCCCGACTTCGTG<br>GTCCGACATCAGCCACATGA	143
MAV_0556	3_F<br>3_R	CATCGAGATCCTCAAGGGGC<br>ATCAGGTCGATCGCCTTGTC	144
MAV_3137	4_F<br>4_R	ACCGGATGCCAAGCTGATC<br>AGAATCGCATAGATGGGGGC	123
MAV_3806	5_F<br>5_R	CGATCGTGCAGTTGGTGTTC<br>TCTTCGACGGCTTGAGTGAC	130
apeB	6_F<br>6_R	AAGGGCTCTGCGAGTTCATC<br>CTCGTCCAGTTCGGTGTACC	101
MAV_1198	7_F<br>7_R	TCAAGAGCGGGTTCTTCGAG<br>TTGAGCTCGCGGATCTTCTC	118
MAV_3367	8_F<br>8_R	GAACTTCAGCAACGCCAAGG<br>GGCATGATCTTGTTCACCGC	105
katG	9_F<br>9_R	GGGGTGAAGAGGACGAATGG<br>GATCAGACCCATGGTGGTGG	129
tuf	10_F<br>10_R	GGTCACGCCGACTACATCAA<br>GACCAGGATGTAGGGCACAC	150
MAV_4925	11_F<br>11_R	GAAGCTGTTGATGCTCAGCG<br>CGTATCCCTCCTTGATCCGC	146
MAV_3858	12_F<br>12_R	TCCCCGGTTCGATTTCCTTG<br>CCGGACCTCCTTGTTGATCC	119

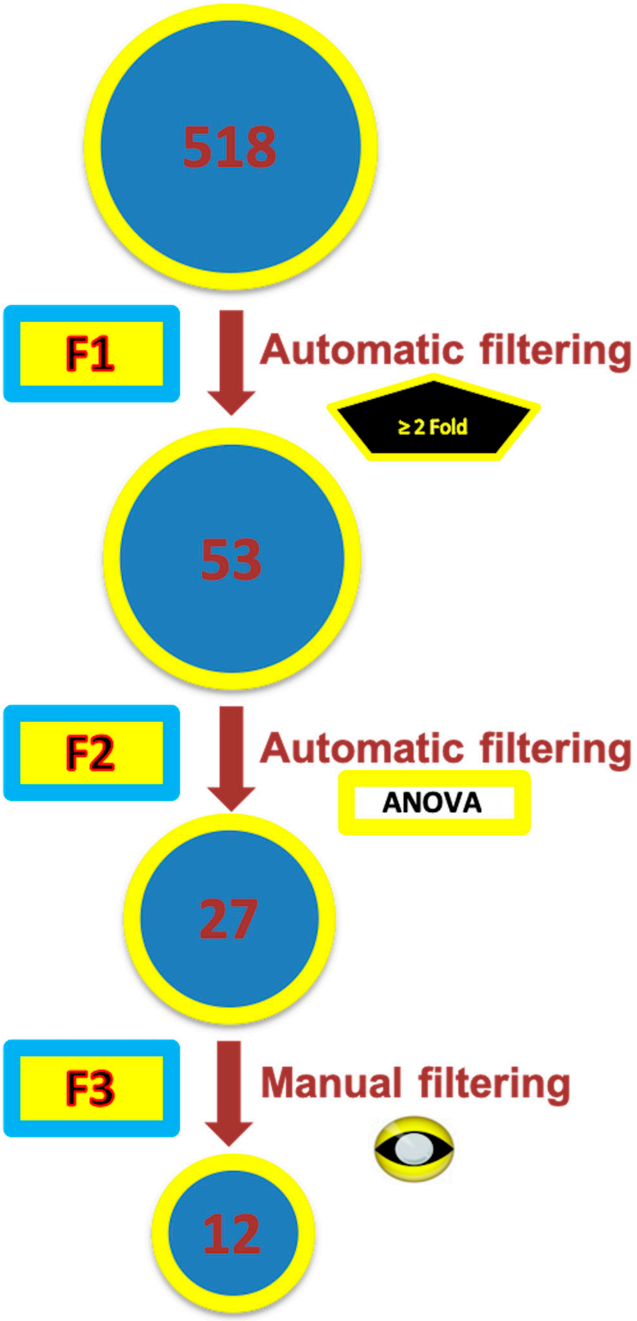

Figure S1: The workflow of our gel spot refining strategy. Three filters (two automated and one manual) were used for refining Progenesis SameSpot selected spots. F1 is a spot volume intensity filter to identify spots different by ≥2-fold. F2 is statistical ANOVA filter to identify spots significantly different at $P \leq 0.05$. F3 is manual filtering to eliminate suspected background and noise spots. Visualization was achieved by silver staining before spot picking and was followed by LC-MS/MS analysis of spots.

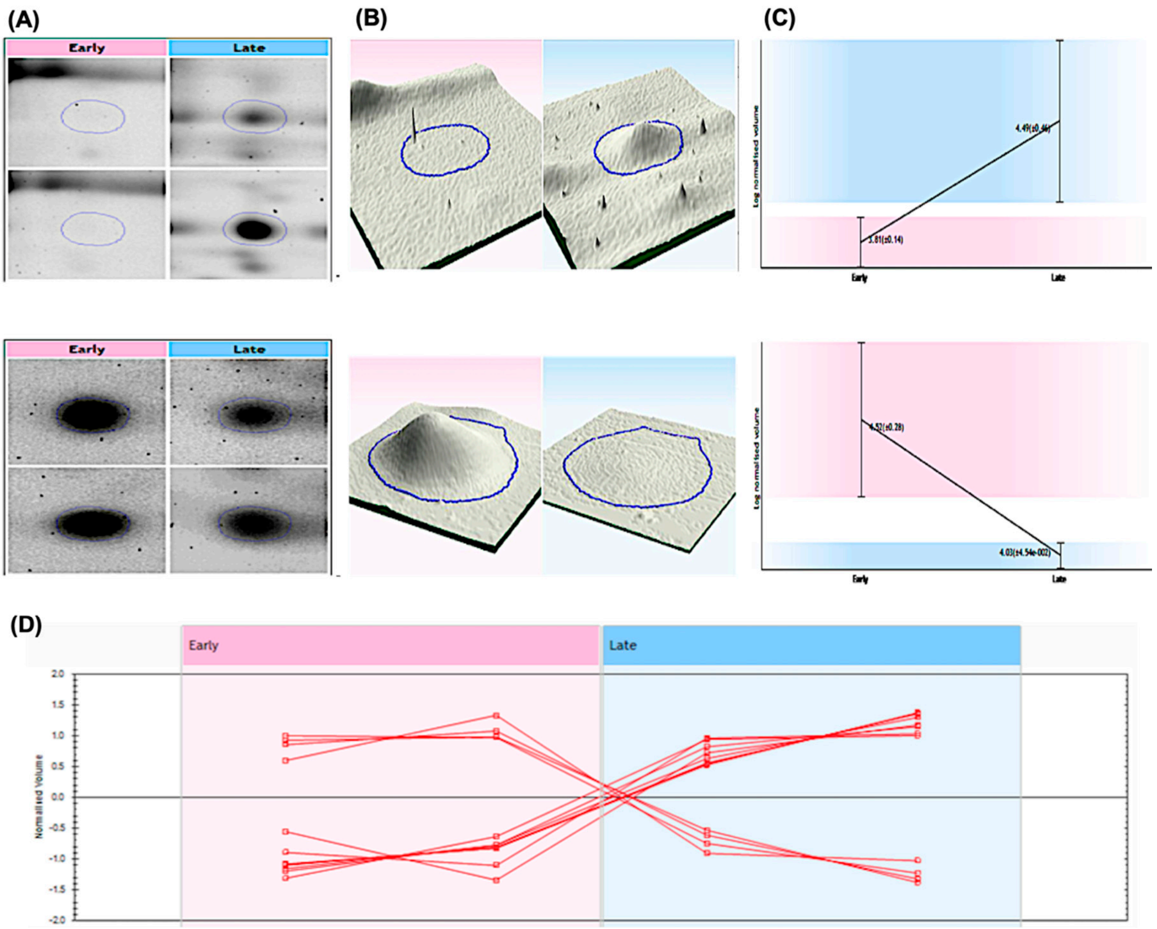

Figure S2: Gel spot comparison for two representatives differentially expressed proteins between the exponential and the stationary growth phases of MAH 104 using Progenesis SameSpots software; Upper line for spot number 63 (Glucose methanol choline) that is up regulated in the stationary phase and lower line for spot number 17 (Universal stress protein) that is down regulated in the stationary phase. Intensities of the two spots differentially expressed in the exponential and the stationary growth phases were shown in the representative gel image (A). 3D views of the intensities of the targeted spots blotted by SameSpots in both phases are shown (B). The logarithmic normalized volume between the 2 phases was blotted for each spot by SameSpots (C). Spot numbers are corresponding to those listed in Table 2. (D) The standard expression profiles of the twelve differentially expressed proteins between the exponential and the stationary growth phases of MAH 104.

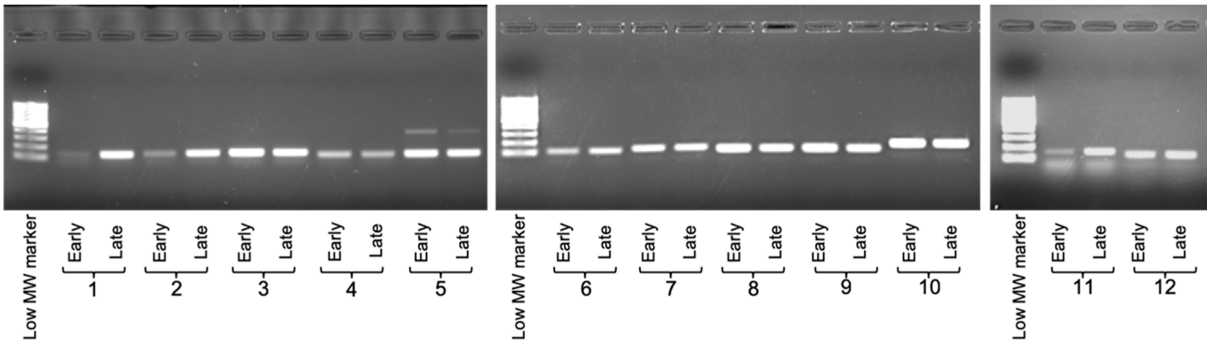

Figure S3: Agarose gel electrophoresis of 12 PCR products of genes responsible for the expression of the 12 differentially expressed proteins between the exponential and the stationary growth phases of *Mycobacteriun avium 104.* Low molecular weight markers are used. Band sizes for each gene product of the 12 genes are summarized in table (1).

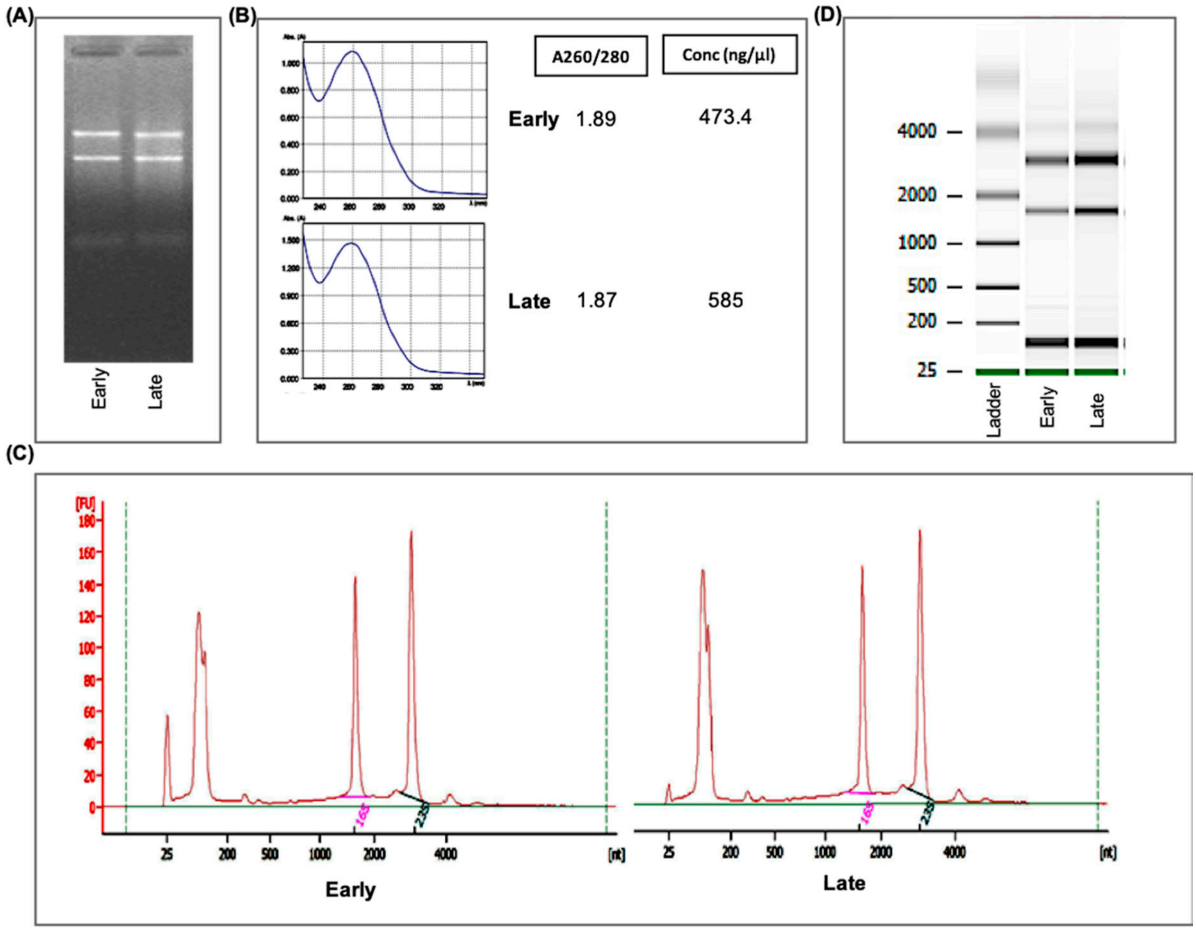

Figure S4: *M. avium* RNA in exponential and in stationary growth phases quality on the gel (A), purity and concentrations using eppendorf Bio-spectrometer (B) and integrity; electropherogram profiles for *M. avium* RNA resolved on a Pico Chip of Agilent 2100 Bioanalyzer, Agilent Technologies (C). (D) Gel image formed for *M. avium* RNA using Agilent 2100 Bioanalyzer, Agilent Technologies.

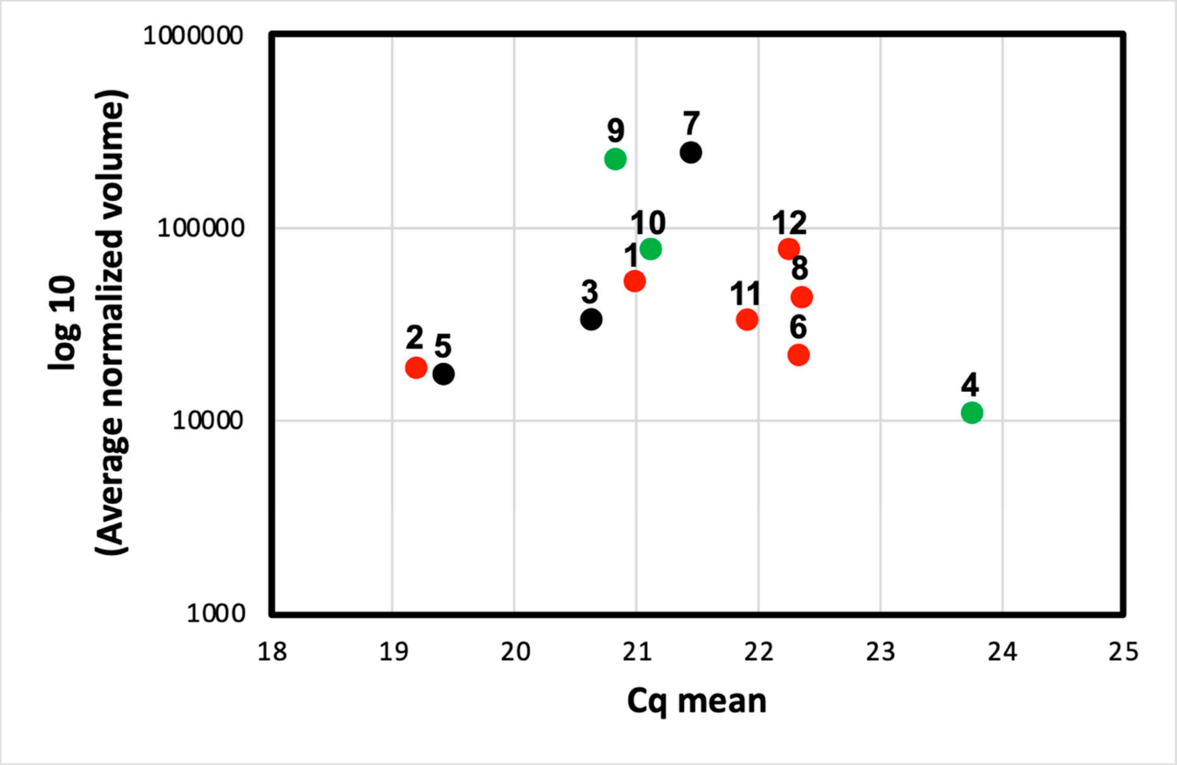

Figure S5: Correlation between the gene expression of each gene and the average normalized volume for each protein of the differentially expressed hits between the two growth phases. Numbers shown here indicating the number of genes and proteins as displayed in table 1.

Red circles; up-regulated, Green circles; down-regulated, Black circles; differ in/between the transcriptomic and the proteomic analysis.
